# 

**Published:** 1870-02

**Authors:** 


Late.—Our Journal again appears too late, owing to circumstances which we have not been able to control. We promise to do better hereafter, and hope this may be a sufficient apology for us this time.

### Books and Pamphlets Received.

Physiology of Man. By Austin Flint, jr., M. D., Professor of Physiology and Microscopy in the Bellevue Hospital Medical College, etc , etc. Volume third: On Secretion; Excretion; Ductless Glands; Nutrition; Animal Heat; Movement; Voice and Speech. New York, D. Appleton & Co. Received through Breed, Lent & Co.

Clinical Lectures on the Principles and Practice of Medicine. By John Hughes Bennett, M. D , F. R. S. E., Professor of the Institutes of Medicine, and Senior Professor of Clinical Medicine in the University of Edinburgh, etc., etc. Fifth American, from the fourth London edition. With five hundred and thirty wood illustrations. New York, Wm. Wood & Co. Received through Breed, Lent & Co.

A Manual of Clinical Medicine and Physical Diagnosis. By Thomas Hawkes Tanner, M. D., F. L. S., etc. Revised and enlarged by Tilbury Fox, M. D., London, Physician to the Skin Department in the University College Hospital. Philadelphia, H. C. Lea Received through T. Butler & Son.

Personal Beauty. How to Cultivate and Preserve it. By D. G. Brinton, M. D., and Geo. H. Naphey's, M. D. Springfield, Mass. W. J. Holland.

Contributions to Practical Laryngoscopy. By A. Ruppaner, M. D., Physician to the New York Dispensary for Diseases of the Throat and Chest.

Remarks on the Galvanic Battery. By G. W. Hough, Director of Dudley Observatory.

Velocity of the Electric Current over Telegraph Wire. By G. W. Hough, A.M,

The Physical and Medical Topography of the City of Wheeling. By James E. Reeves, M. D.

Sixteenth Annual Report of the Trustees of the State Lunatic Hospital at Taunton

Second Annual Report of the New York Ophthalmic Dispensary, located at 1299 Broadway.

Commencement of the Regular Spring Course of Practical Instruction at the Medical College of Ohio, at Cincinnati.

Insurance Monitor.

